# Primary Fermentation in Wine Production Influence on Phenolic Retention and Valorization Potential of Berry Skin By-Products

**DOI:** 10.3390/plants15020296

**Published:** 2026-01-19

**Authors:** Audrone Ispiryan, Elvyra Jarienė

**Affiliations:** Bioeconomy Research Institute, Agriculture Academy, Vytautas Magnus University, Studentu Str. 11, LT-53361 Akademija, Lithuania; elvyra.jariene@vdu.lt

**Keywords:** berry by-products, fermentation, phenolic composition, primary wine fermentation, valorization, functional plant materials, antioxidant capacity

## Abstract

Berry skins are rich in phenolic compounds but are commonly discarded as low-value waste during berry wine production. The present study evaluated how primary alcoholic fermentation affects the retention and transformation of phenolics in berry skins of blackcurrant (*Ribes nigrum* L.), black chokeberry (*Aronia melanocarpa* L.), lingonberry (*Vaccinium vitis-idaea* L.), rowanberry (*Sorbus aucuparia* L.), and cranberry (*Vaccinium macrocarpon* L.). Non-fermented and fermented skin fractions were analysed using Folin–Ciocalteu and HPLC to determine total and individual phenolic profiles. Primary fermentation induced significant species-dependent changes in phenolic composition. Blackcurrant, lingonberry, and rowanberry skins exhibited substantial decreases in total phenolics (−66%, −26%, and −57%, respectively), driven by strong losses of flavan-3-ols and hydroxycinnamic acids. In contrast, cranberry and chokeberry skins showed net increases in phenolic content (+47% and +18%, respectively), associated with the release of bound phenolics and the appearance of new low-molecular-weight phenolic acids such as gallic acid. Across all species, fermentation enhanced biotransformation into simpler phenolics while reducing major native anthocyanins and catechins. These results demonstrate that the influence of primary fermentation on berry skins is not uniform but dictated by their inherent phenolic architecture. Berries rich in polymeric or conjugated phenolics benefit from fermentation through increased phenolic extractability. The findings provide a comparative basis for optimizing fermentation and post-processing strategies to enhance the valorization potential of berry by-products in food and nutraceutical applications.

## 1. Introduction

Polyphenols are widely studied phytochemicals due to their health-protective effects as antioxidants [1], anti-inflammatory agents [2], and anti-diabetic agents [3,4,5,6,7], and the global demand for natural polyphenols is rising. The market is projected to reach ~USD 2.1 billion by 2025 [8]. Berries are among the richest sources of dietary polyphenols and exhibit high antioxidant capacities. The present study focuses on five berry species—black currant (*Ribes nigrum* L.), black chokeberry (aronia, *Aronia melanocarpa* L.), lingonberry (*Vaccinium vitis-idaea* L.), rowanberry (*Sorbus aucuparia* L.), and cranberry (*Vaccinium macrocarpon* L.)—selected for their regional abundance and diverse phenolic profiles. These particular berries are well known in northern climates and have each been reported to contain a broad spectrum of phenolic constituents. Black currant and aronia fruits are especially rich in anthocyanin pigments, alongside other flavonoids and phenolic acids, contributing to their potent antioxidant activity [9,10,11,12,13,14]. Lingonberry and cranberry are characterized by high levels of proanthocyanidins, flavonols, and benzoic and hydroxycinnamic acids [15,16,17,18,19]. Rowan berries also provide significant polyphenol content and antioxidant capacity, though they are less commonly utilized as food due to their bitterness and astringency [20,21,22]. All these berries have bioactivities associated with their phytochemicals, which underpins the interest in further valorizing their constituents for health-promoting applications.

When berries are processed for juice, wine, or other products, the solid berry skins are largely discarded despite being a concentrated source of phytochemicals. Depending on the fruit, 20–60% of the raw berry weight remains as pomace (skins, seeds, pulp) after pressing. This pomace harbors substantial amounts of polyphenols that do not transfer to the juice. Indeed, it is estimated that a significant fraction of a berries’s total polyphenols can reside in the solid residues. Roughly 28–35% of polyphenols may remain in the skins (and ~60–70% in seeds), versus only ~10% in the juice-extracted pulp. These berry skins and seeds are, therefore, a potentially valuable source of natural antioxidants [23,24,25,26,27].

Many berry-processing by-products are still treated as waste and utilized only in low-value applications, resulting in the loss of both bioactive compounds and economic potential [28,29,30]. At the same time, demand for innovative and sustainable products is rapidly increasing, yet small and medium-sized producers seldom exploit these resources due to limited access to knowledge from a scientific point of view [31,32,33]. Although studies confirm that berry skins and pomaces are rich in phenolic compounds with diverse biological activities, these materials remain insufficiently investigated, particularly berry skins, which contain highly concentrated pigments and phytochemicals.

Berry skins remaining after juice extraction contain a considerable share of phenolic compounds [34], yet they are sensitive to spoilage and quality loss due to high moisture and residual sugars. When these materials are subjected to primary alcoholic fermentation, as commonly done in fruit wine production, the biochemical environment around phenolic compounds changes. Yeast activity may disrupt the plant cell–matrix and promote the conversion of bound phenolics into more extractable forms. At the same time, some compounds, particularly pigments or easily oxidised phenolics, can degrade or diffuse into the surrounding fermenting liquid. The final effect on the solid berry fraction, therefore, varies depending on the species and the structural organization of their phytochemicals.

During fruit wine production, technological parameters such as yeast selection, the use of enzymatic preparations, fermentation temperature, and maceration duration play a critical role in determining the fate of phenolic compounds in both the liquid phase and the resulting pomace. Previous studies have demonstrated that these processing variables can significantly influence phenolic extraction, degradation, and biotransformation, thereby affecting the antioxidant and antiradical properties of fermentation by-products. In particular, variations in yeast metabolism and maceration conditions have been shown to alter the retention and composition of phenolic compounds remaining in pomace after fermentation, highlighting the importance of process design for by-product quality and valorization potential [35].

A systematic comparison of fermented and non-fermented skins can provide important insight into how fermentation influences the retention, transformation and accessibility of phenolic constituents that remain in the solid phase. Such knowledge is essential when considering berry processing residues as a potential source of natural antioxidants or as functional ingredients in food and nutraceutical formulations. Understanding these species-specific differences will also help determine whether fermentation contributes positively to the valorization of berry by-products or if alternative processing strategies would be more suitable.

The present study investigates the phenolic composition of non-fermented and fermented berry skins from five commonly used berry species. The analysis focuses on berry skins as the most polyphenol-rich yet frequently discarded portion of the fruit. In contrast to most previous studies, which primarily examine whole fruits, juices, or total pomace, this study specifically isolates and investigates the skin fraction obtained after industrially relevant separation steps, and, by comparing multiple berry species processed under identical fermentation conditions, the work provides a systematic evaluation of species-specific phenolic transformations within the skin matrix, offering insights that are directly applicable to by-product valorization strategies.

Primary fermentation is expected to influence the abundance and profile of phenolic compounds in a manner that depends on the species and the structural organization of their phytochemicals. In some berries, the release of bound phenolics may increase the measurable content in the solid fraction, whereas in others, sensitive compounds such as anthocyanins may undergo partial degradation during fermentation. Clarifying these differences will indicate whether fermentation enhances the functional value of berry skins and contributes to their potential use as natural antioxidant sources or other value-added ingredients, supporting both waste reduction and the development of sustainable products in plant-based processing chains.

## 2. Research Methods

### 2.1. Sample Preparation

Five berry species were selected as raw materials for the study: black currant (*Ribes nigrum* L.), black chokeberry (*Aronia melanocarpa* L.), lingonberry (*Vaccinium vitis-idaea* L.), rowanberry (*Sorbus aucuparia* L.) and cranberry (*Vaccinium macrocarpon* Aiton). The fruits used in this study were commercially cultivated berry varieties commonly grown in the Baltic region (Lithuania). Specific cultivar identification was not performed, as the study was designed as applied research focusing on species-level responses and processing-related phenolic transformations relevant to industrial berry by-products. The use of commercially available fruits without cultivar differentiation reflects realistic processing conditions and supports the applicability of the findings to practical valorization strategies. Fruit ripeness was determined based on commercial harvest maturity, assessed using a combination of visual color development, firmness, and soluble solids content, following standard agricultural practices for each berry species. All fruits were harvested at full technological maturity suitable for processing.

The berries were grown and harvested under temperate climatic conditions, and supplied by local producers during the same harvest season to minimize environmental and seasonal variability. After harvest, the fruits were rapidly frozen at −29 °C to preserve their native composition until further processing. For each species, two types of samples were prepared: skins obtained after juice pressing (non-fermented) and skins collected following primary alcoholic fermentation, enabling direct comparison of phenolic characteristics between both treatments.

### 2.2. Primary Fermentation

The initial soluble solids content of the fresh berry fruits, measured prior to processing, ranged from 9.2 to 12.8 °Brix, depending on the berry species. The berries were crushed to a pulp (including skins, pulp, and seeds) and prepared for primary fermentation. To standardize fermentation conditions, sucrose was added to all berry mashes to achieve a final soluble solids content of 19.0 °Brix before inoculation. Alcoholic fermentation proceeded until completion, resulting in a final ethanol concentration ranging from 9.5 to 11.2 vol%, depending on the berry matrix. Fermentation was allowed to proceed until completion, thereby minimizing the influence of initial sugar differences on the final phenolic composition.

Fermentation was carried out in food-grade fermentation vessels at 21 °C for 14 days. A commercial active dry yeast (*Saccharomyces cerevisiae* strain) Oenoferm^®^ Universal (Erbslöh, Geisenheim, Germany) was used as the starter culture to inoculate the mash. Yeast was added according to the manufacturer’s instructions (approximately 20–30 g per 100 L of mash). After inoculation, the mixture was stirred, sealed with an airlock, and every two days the fermenting mash was thoroughly mixed to ensure uniform fermentation and prevent sedimentation. Fermentation progress was monitored daily by observing CO_2_ release and measuring density changes. After 14 days, the process was considered complete, indicated by cessation of CO_2_ evolution and stabilization of the specific gravity.

### 2.3. Skins Separation and Drying

Berry skins were obtained from fermented/non-fermented berry mash after solid–liquid separation by centrifugation (Voran, Pichl bei Wels, Austria). The resulting pomace had a loose structure, which allowed further fractionation by sieving. Seeds were removed using a fine mesh sieve, and the remaining fraction consisted predominantly of berry skins, which were used for subsequent analyses. After separation, the berry skins were dried at 35 °C in a low-temperature infrared dryer (InfraRed-32, Ukrsushka, Dnepr, Ukraine). Drying in both cases was continued until the seed moisture content reached 9–12%, which was confirmed using a moisture analyzer (halogen lamp drying principle, measuring until stable mass was achieved) (MA 35, Sartorius, Göttingen, Germany). Once dried, the material was sieved through a fine mesh (aperture 1–2 mm) to separate the seeds from berry skins and other residues. This gentle drying approach (below 40 °C) minimized thermal degradation of the seed oils and bioactive compounds. The skins obtained from both groups were cooled to ambient temperature, sealed in air-tight containers, and stored in the dark until oil extraction.

### 2.4. Reagents

Analytical and HPLC-grade reagents and solvents were used for all chemical analyses. Folin–Ciocalteu reagent, sodium carbonate and aluminum chloride were obtained from Sigma-Aldrich (Steinheim, Germany). Standards of phenolic acids and flavonoids used for HPLC identification and quantification, including gallic acid, chlorogenic acid, caffeic acid, p-coumaric acid, quercetin-3-O-rutinoside, quercetin-3-O-glucoside, kaempferol-3-O-glucoside, catechin and myricetin, were also purchased from Sigma-Aldrich. Methanol and acetonitrile (HPLC grade), as well as trifluoroacetic acid and ortho-phosphoric acid used for mobile phase preparation, were acquired from Fluka Chemika (Buchs, Switzerland). Ethanol (analytical grade) was used for the preparation of DPPH radical solutions. Ultrapure deionized water (18.2 MΩ·cm) required for extraction and spectrophotometric assays was produced using a Millipore Synergy purification system (Bedford, MA, USA). All reagents were stored and handled according to the manufacturers’ recommendations to ensure stability and reliability of analytical results.

### 2.5. Total Phenolic Compounds

Total phenolic content was determined using the Folin–Ciocalteu method, following the procedure described by Singleton and Rossi (1965) with minor modifications [36]. Briefly, 0.2 mL of extract was mixed with 1.0 mL of Folin–Ciocalteu reagent (previously diluted 1:10 with distilled water) and 0.8 mL of sodium carbonate solution (7.5%, *w*/*v*). The reaction mixture was incubated at room temperature in the dark for 30 min, and absorbance was measured at 765 nm using a UV-Vis spectrophotometer (Onda V-10 Plus, Ignara, Vilnius, Lithuania). The results were calculated based on a gallic acid calibration curve and expressed as mg gallic acid equivalents per 100 g of dry weight (mg GAE/100 g DW).

### 2.6. Antioxidant Activity

Antioxidant activity was determined using the DPPH radical scavenging assay according to Brand-Williams et al. (1995) [37] with minor modifications. Briefly, 0.1 mL of berry skin extract was mixed with 3.9 mL of freshly prepared DPPH solution (0.1 mM in ethanol). The reaction mixture was incubated in the dark at room temperature for 30 min, and absorbance was measured at 515 nm using a UV–Vis spectrophotometer (Onda V-10 Plus, Ignara, Vilnius, Lithuania). Trolox was used as a reference standard, and results were expressed as µmol Trolox equivalents per gram of dry weight (µmol TE/g DW). All measurements were carried out in triplicate, and mean values with standard deviations are reported.

### 2.7. Identification and Quantification of Phenolic Compounds

Phenolic compounds were identified and quantified using high-performance liquid chromatography (HPLC) (Shimadzu, Kyoto, Japan). Chromatographic separation was performed on a reversed-phase C18 column (250 × 4.6 mm i.d., 5 µm particle size). Prior to chromatographic analysis, berry skin extracts were filtered through 0.22 µm PVDF membrane filters to remove suspended particles and ensure reliable separation. The mobile phase consisted of acidified water and acetonitrile, delivered by a gradient elution program optimized for separation of phenolic acids, flavonoids and flavan-3-ols. The flow rate was kept constant at 1.0 mL/min throughout the analysis. A gradient elution was applied using water with 0.1% formic acid (A) and acetonitrile (B), with a total run time of 60 min per sample.

Detection was carried out using a UV–Vis diode-array detector by continuously recording absorbance spectra in the range relevant for phenolic compounds. Quantification was based on absorbance at characteristic wavelengths according to compound class: 280 nm for flavan-3-ols, 320 nm for phenolic acids and 360 nm for flavonols. Identification of analytes was achieved by comparing retention times and UV spectral data with those of authenticated reference standards. External calibration curves for each target compound, prepared from serial dilutions of the standards, were used to determine the concentrations in the extracts. Results were expressed as milligrams per gram of dry weight (mg/g DW).

All experiments were performed in three replicates (*n* = 3). Chromatographic data were processed using the instrument manufacturer’s software, and peak areas were used for quantitative evaluations. The method ensured high analytical repeatability and allowed reliable comparison of phenolic profiles between non-fermented and fermented berry skin samples. Chromatograms supporting results are available in the Supplementary Materials.

### 2.8. Statistical Analysis

All the experiments were carried out in triplicate. In the statistical processing of the data obtained from the analysis of the chemical composition of the fruits, the standard deviation was calculated and presented together with the mean values. Microsoft Excel 2019 (Microsoft Corporation, Redmond, WA, USA) and IBM SPSS Statistics 26 (IBM Corp., Armonk, NY, USA) software packages were used for statistical analysis. One-way analysis of variance (ANOVA), along with the post hoc Tukey’s HSD test, was employed for statistical analysis. Differences were considered to be significant at *p* < 0.05. The antioxidant activity was evaluated using the DPPH assay.

To explore relationships among antioxidant activity in berry skins, multivariate statistical analyses were applied. Data for tocopherols (mg/kg), total phenolic content (mg GAE/100 g), DPPH radical scavenging activity (% inhibition), and oxidative stability (h) were first averaged from three independent replicates and then standardized by Z-score normalization prior to analysis.

A hierarchical clustering heatmap was generated using Euclidean distance and Ward’s linkage to group samples according to similarities in their biochemical profiles. This approach enabled simultaneous comparison of fermented (F) and non-fermented (NF) berry skins across species and highlighted co-variation among the measured variables.

For dimensionality reduction and visualization of overall variance, principal component analysis (PCA) was performed on the same normalized dataset. The first two principal components (PC1 and PC2) were extracted and plotted to display sample clustering (score plot) and variable contributions (loading plot). Score plots differentiated berry species and treatment conditions, while loading plots indicated the relative influence of tocopherols, phenolics, DPPH activity, and oxidative stability on the principal components. All analyses and visualizations were performed in Python 3.11 (Python Software Foundation, Wilmington, DE, USA) using the libraries scikit-learn 1.4, pandas 2.2, seaborn 0.13, and matplotlib 3.8.

## 3. Results

**Figure 1:**
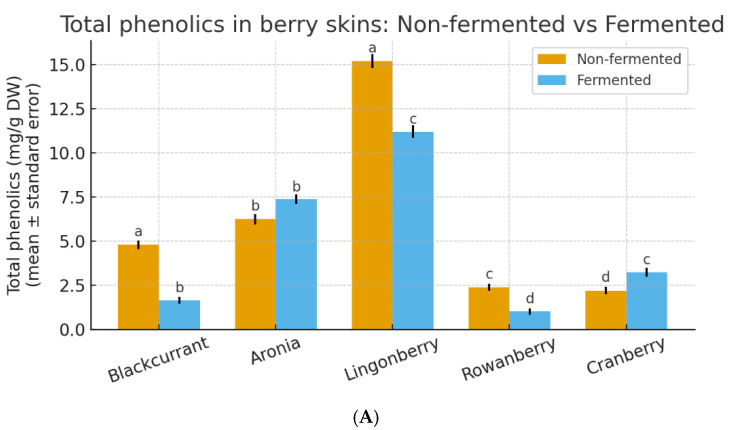

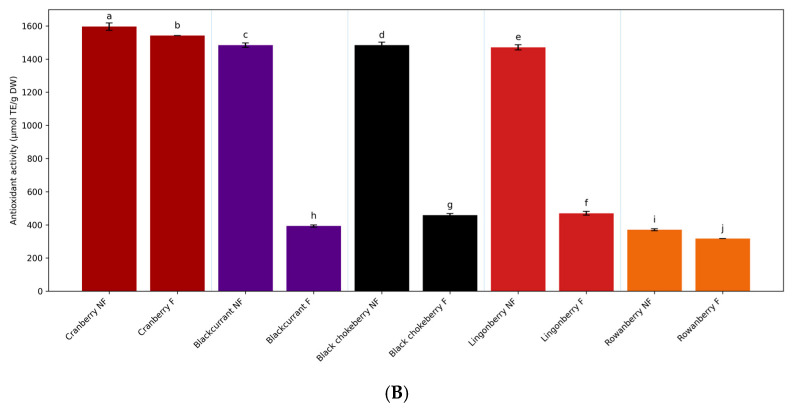
Total phenolics (**A**) and antioxidant activity (**B**) in berry skins: Non-fermented vs. Fermented. Note: Values are presented as mean ± standard error (*n* = 3). Different lowercase letters above the bars indicate statistically significant differences among all samples included in the analysis, as determined by one-way ANOVA followed by Tukey’s HSD post hoc test applied across both non-fermented and fermented samples (*p* < 0.001).

To evaluate how primary fermentation affected the retention and availability of polyphenols in the solid fraction, total phenolic content (A) and antioxidant activity (B) were quantified in non-fermented and fermented berry skins across all five species, as shown in Figure 1 below. The obtained values revealed clear species-specific differences in both the baseline phenolic levels and their responses to fermentation.

Primary fermentation differently affected total phenolic content across species. Primary fermentation had distinct effects on the total phenolic content depending on the berry species. Blackcurrant skins exhibited the strongest decrease (4.79 to 1.64 mg/g DW, −66%), revealing a substantial loss of easily extractable phenolics. Considering the known prevalence of anthocyanins and flavan-3-ols in blackcurrant skins, this decline likely reflects oxidative and enzymatic degradation of these compounds, as well as their diffusion from the solid matrix into the fermentation medium. A similar, although less pronounced, reduction was observed in lingonberry (15.18 to 11.19 mg/g DW, −26%). These berries contain a high proportion of catechin and p-coumaric acid, which sharply decreased in fermented samples, suggesting that microbial metabolism and chemical instability outweighed any enzymatic release of bound phenolics. Rowanberry skins showed a marked decline as well (2.38 to 1.02 mg/g DW, −57%), indicating limited structural protection of phenolic acids and a relatively small pool of conjugated phenolics that could be liberated during fermentation.

In contrast, Aronia and cranberry demonstrated an increased availability of phenolics following fermentation. Aronia skins exhibited a moderate rise (6.24 to 7.37 mg/g DW, +18%), likely linked to the release of bound flavonols such as kaempferol derivatives, despite partial anthocyanin degradation. Cranberry showed the largest relative increase (2.20 to 3.24 mg/g DW, +47%), consistent with the enzymatic depolymerization of proanthocyanidins and the emergence of low-molecular-weight phenolic acids (e.g., gallic acid) that became newly detectable after fermentation. Rowanberry exhibited only a minor decline (≈2.3 to 2.1–1.6 mg/g DW), reflecting a limited pool of bound phenolics and moderate degradation of existing hydroxycinnamic acids. The net change in total phenolics in all species depended on the balance between degradation of native anthocyanins and hydroxycinnamates versus liberation of bound forms into extractable simple phenolics.

Antioxidant activity of berry skins measured by the DPPH assay showed clear, quantitative correspondence with total phenolic content. Among non-fermented samples, cranberry skins exhibited the highest antioxidant capacity (1596 ± 22 µmol TE/g DW), followed by blackcurrant (1484 ± 13 µmol TE/g DW), black chokeberry (1484 ± 18 µmol TE/g DW), and lingonberry (1471 ± 16 µmol TE/g DW), reflecting their similarly high phenolic concentrations. Rowanberry skins displayed substantially lower antioxidant activity (370 ± 6 µmol TE/g DW), in agreement with their lower total phenolic content.

Fermentation induced pronounced but species-specific changes in antioxidant capacity. In blackcurrant skins, antioxidant activity decreased by approximately 74%, from 1484 ± 13 to 393 ± 7 µmol TE/g DW, closely paralleling the strong reduction in total phenolics observed after fermentation. Similar relative decreases were recorded for lingonberry (from 1471 ± 16 to 470 ± 11 µmol TE/g DW) and rowanberry skins (from 370 ± 6 to 317 µmol TE/g DW). In contrast, fermented cranberry skins retained very high antioxidant activity (1542 µmol TE/g DW), remaining close to the non-fermented level, which is consistent with the preservation of a high phenolic pool dominated by low-molecular-weight phenolic acids.

The data presented in Table 1 show that, in total, 23 phenolic compounds were identified in the skins of black currant, aronia, lingonberry, rowanberry, and cranberry, both in their non-fermented and fermented forms. The most characteristic phenolics across all samples were hydroxycinnamic acids (chlorogenic, caffeic, and p-coumaric acids), flavan-3-ols (catechin, epigallocatechin), and flavonols (quercetin and kaempferol derivatives). Among these, catechin and p-coumaric acid were dominant in lingonberries, while black currant and aronia were rich in flavonols such as quercetin-3-O-glucoside and kaempferol. Cranberries were distinguished by the presence of gallic and ellagic acids, which increased following fermentation. Fermentation markedly shifted the phenolic profile in most berries, generally reducing flavan-3-ols but elevating simple phenolic acids such as benzoic and ellagic acids. These changes emphasize the role of fermentation in modifying the availability and relative contribution of different phenolic classes within berry skins.

Results reveal that black currant skins contain a diverse phenolic profile, with sinapic acid (1.32 ± 0.01 µg/g DW) and p-coumaric acid (0.99 ± 0.01 µg/g DW) as the most abundant phenolic acids in the unfermented (juice-pressed) skins. In fact, catechin was identified as the major phenolic in currants. Our data confirm that sinapic acid is highest in black currant (NF), followed by p-coumaric acid and benzoic acid (0.51 ± 0.00 µg/g DW). After fermentation, most of these compounds significantly decreased: sinapic acid content fell to 0.28 ± 0.01 µg/g DW, and p-coumaric acid was almost completely depleted (to 0.00 µg/g DW), indicating a large reduction. Benzoic acid content also dropped from 0.51 to 0.23 µg/g DW, and catechin content declined from 0.35 to 0.11 µg/g DW. By contrast, chlorogenic acid content showed a slight increase (from 0.15 to 0.19 µg/g DW). Other minor acids (caffeic, vanillic) remained at very low concentrations. Fermentation greatly reduced key cinnamic acids in black currant skins, while some acids were stable or slightly enriched. These findings align with the known high phenolic content of black currants, where anthocyanins and flavan-3-ols dominate [38,39].

Aronia berries are among the richest sources of polyphenols. In our unfermented Aronia skins, the most abundant compounds were salicylic acid content (1.81 ± 0.00 µg/g DW) and the flavonol kaempferol content (1.92 ± 0.01 µg/g DW). Caffeic acid content (0.19 ± 0.00 µg/g DW) and catechin content (0.21 ± 0.00 µg/g DW) were also present at moderate levels. After fermentation, Aronia skins showed a notable increase in kaempferol content (up to 2.98 ± 0.01 µg/g DW) and its glycoside kaempferol-3-O-glucoside content (0.26 to 0.95 µg/g DW), while decreases occurred in salicylic acid content (1.81 to 1.06 µg/g DW) and catechin (0.21 to 0.00 µg/g DW) contents. Caffeic acid content approximately doubled (0.19 to 0.43 µg/g DW), and p-coumaric acid content after fermentation (0.00 to 0.59 µg/g DW). Benzoic acid rose from 0.02 to 0.15 µg/g DW. Fermentation of Aronia skins tended to shift the profile from phenolic acids toward higher flavonol content. These results are consistent with Aronia’s high phenolic nature: chokeberries are rich in anthocyanins and phenolic acids, though lower in flavonols. Here we see fermentation enhancing kaempferol levels, possibly by releasing bound forms [40,41,42].

Lingonberries are known to be abundant in catechin and organic acids. In unfermented lingonberry skins, catechin (5.13 ± 0.03 µg/g DW) was by far the compound with the highest concentration, followed by p-coumaric acid (4.35 ± 0.79 µg/g DW) and valeric acid (3.85 ± 0.03 µg/g DW) contents. These three compounds dominated the profile. After fermentation, catechin dropped sharply (5.13 to 1.31 µg/g DW) and p-coumaric acid decreased (4.35 to 1.68 µg/g DW), indicating extensive loss into the wine. Valeric acid also declined (3.85 to 2.88 µg/g DW). In contrast, several other compounds increased in fermented lingonberry skins: salicylic acid rose from 0.16 to 0.84 µg/g DW, sinapic acid from 0.08 to 0.43 µg/g DW, and trans-cinnamic acid appeared (0.00 to 1.01 µg/g DW). Benzoic acid content modestly increased (0.09 to 0.53 µg/g DW). Fermentation transformed the lingonberry profile: the dominant hydroxycinnamates were reduced, while various hydroxybenzoic acids and flavonols were enhanced. This is in line with reports that catechin is a major contributor to lingonberry composition [43].

Rowanberry skins contained moderate levels of certain phenolics in the NF state. The dominant phenolic was caffeic acid (0.50 ± 0.00 µg/g DW), along with notable salicylic acid (0.65 ± 0.00 µg/g DW) and a flavonoid, quercetin-3-O-glucoside (0.54 ± 0.00 µg/g DW). Ellagic acid (0.13 µg/g DW) and apigenin (0.07 µg/g DW) were detected only in rowanberry samples. After fermentation, all of these compounds decreased substantially: caffeic acid fell to 0.20 µg/g DW, salicylic acid to 0.30 µg/g DW, and quercetin-3-glucoside to 0.20 µg/g DW. Apigenin halved (0.07 to 0.03 µg/g DW). One exception is sinapic acid, which slightly increased (0.03 to 0.09 µg/g DW). Fermentation led to the consumption of many rowan phenolics (glycosides and acids). This pattern supports the notion that microbial fermentation hydrolyzes glycosidic phenolics into simpler forms [44,45].

Cranberries are also rich in flavonols and phenolic acids. Analysis results show that in unfermented cranberry skins, the largest compounds were the flavonol glycosides quercetin-3-O-glucoside (0.50 ± 0.02 µg/g DW) and quercetin-3-O-rutinoside (0.40 ± 0.02 µg/g DW). Catechin (0.50 µg/g DW) and ellagic acid (0.30 µg/g DW) contents were moderate. Notably, gallic acid and several other acids were essentially absent pre-fermentation. Following fermentation, however, gallic acid appeared at 1.96 ± 0.01 µg/g DW (the highest value in any cranberry sample), consistent with glycoside hydrolysis during fermentation. Quercetin-3-O-glucoside decreased to 0.16 and quercetin-3-O-rutinoside to 0.29 µg/g DW. Ellagic acid decreased to 0.19 µg/g DW. Meanwhile, minor acids such as vanillic (0.03), salicylic (0.08), sinapic (0.09), and trans-cinnamic (0.10) emerged after fermentation. These results indicate that fermentation drastically altered the cranberry skin profile: many flavonol glycosides were depleted, while several simple phenolic acids appeared, notably gallic acid.

In all five berries, the dominant phenolics in the unfermented skins were large flavonoids or acids (e.g., catechin in lingonberry; sinapic and p-coumaric acids in black currant; kaempferol in Aronia) with concentrations ranging from ~0.5 to 5 µg/g DW. After primary fermentation, these compounds generally decreased (often significantly), while simpler phenolic acids or free aglycones increased, reflecting glycoside hydrolysis and extraction processes. Fermentation greatly reduced the catechin content of lingon and eliminated p-coumaric in black currant, whereas it generated substantial gallic acid in cranberry skins. The most abundant compounds in each berry thus shifted: black currant was richest in sinapic acid (NF) and benzoic/catechin (F), Aronia in kaempferol (F) and salicylic (NF), lingonberry in catechin (NF) and quercetin-3-glucoside (F), rowan in caffeic (NF) and salicylic acids, and cranberry in quercetin glycosides (NF) and gallic acid (F).

**Figure 2:**
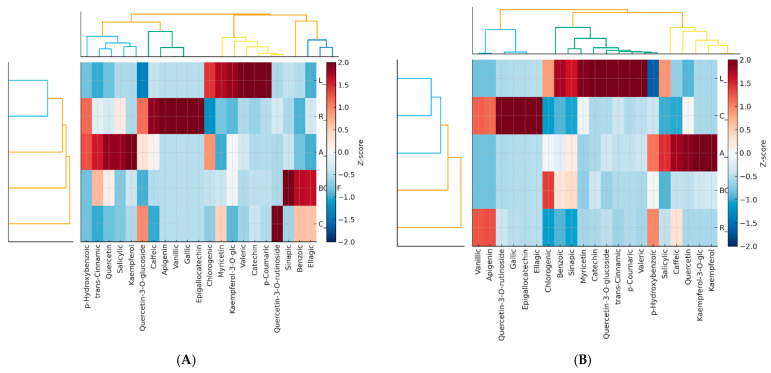
Heatmap of individual phenolic compounds in non-fermented (**A**) and fermented berry (**B**) skins. Note: Sample codes represent berry species and processing state as follows: BC-NF, black currant non-fermented; BC-F, black currant fermented; A-NF, Aronia non-fermented; A-F, Aronia fermented; L-NF, lingonberry non-fermented; L-F, lingonberry fermented; R-NF, rowanberry non-fermented; R-F, rowanberry fermented; C-NF, cranberry non-fermented; C-F, cranberry fermented. Hierarchical clustering of the same 23 phenolic compounds after primary fermentation. Data are Z-score standardized to show relative enrichment or reduction following fermentation. Rows and columns clustered using Ward’s linkage and Euclidean distance.

To further explore species-specific differences in phenolic profiles and how they were affected by fermentation, hierarchical cluster heatmaps were constructed for both non-fermented and fermented berry skins (Figure 2 below). Data were Z-score standardized to visualize relative dominance or depletion of each compound within a species, while clustering enabled grouping of berries and phenolic compounds according to their similarity patterns. This approach highlights not only abundance changes but also which phenolic subclasses are most responsive to primary fermentation.

Results reveal that, in the non-fermented samples, lingonberry skins formed a distinct cluster driven by very high concentrations of catechin, p-coumaric acid, and valeric acid. Aronia and cranberry displayed closer relatedness, characterized by elevated flavonols, whereas blackcurrant and rowanberry were grouped together due to their predominantly hydroxycinnamic acid profiles. Fermentation markedly altered these relationships. In fermented skins, cranberry and lingonberry clustered together, reflecting a shift toward simple phenolic acids generated by enzymatic depolymerization of complex tannins and conjugates. Aronia retained a unique position due to high levels of kaempferol and its glycosides, suggesting enhanced release of bound flavonols. Conversely, blackcurrant and rowanberry were separated from other species through overall reduction in major phenolic groups, consistent with extensive degradation of anthocyanins and hydroxycinnamates.

Across both datasets, hierarchical clustering effectively illustrates the two dominant biochemical outcomes of fermentation: (i) degradation-driven loss in anthocyanin-rich matrices (blackcurrant, rowanberry), and (ii) liberation-driven increases in berries rich in conjugated phenolics (cranberry, aronia, partly lingonberry). These observations confirm that the direction of fermentation effects is determined by each berry’s inherent phenolic composition and structural organization, with hydrolysis and oxidative metabolism acting as competing processes.

**Figure 3:**
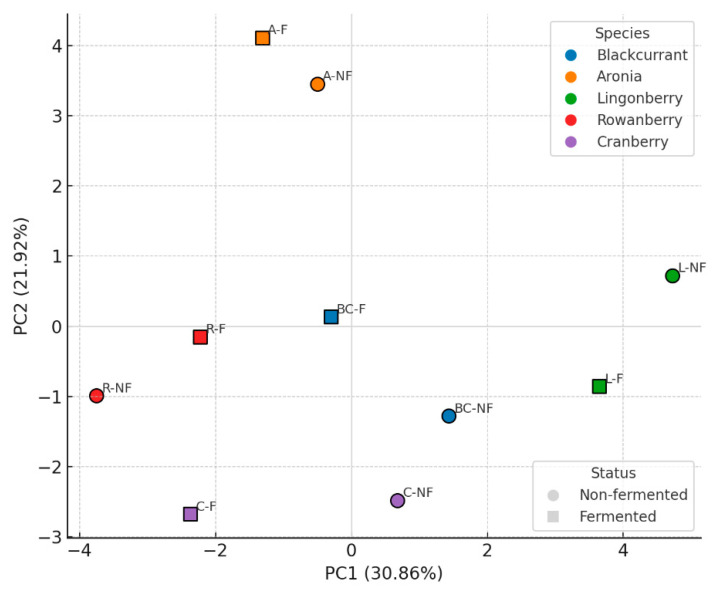
PCA score plot of phenolic profiles in berry skins.

To explore how fermentation modified the phenolic composition of berry skins, principal component analysis (PCA) was performed on the quantified phenolics. The first two principal components explained over half of the variance in the dataset (PC1 = 30.86%, PC2 = 21.92%), revealing clear species-level separation (Figure 3 below).

The PCA score plot showed that non-fermented samples from each berry species were spatially distinct, confirming characteristic phenolic fingerprints. Fermentation shifted all samples in the PCA space, but the magnitude and direction of this shift differed by species. Lingonberry exhibited the most pronounced displacement along PC1, indicating substantial modification of its phenolic profile. Aronia samples also shifted noticeably toward higher PC2 scores following fermentation. In contrast, blackcurrant and rowanberry underwent smaller shifts, suggesting a more conserved phenolic composition. Cranberry moved toward lower PC2 values, reflecting selective release or transformation of specific compounds. The PCA highlights that fermentation induces species-dependent restructuring of phenolic profiles in berry skins, supporting the quantitative findings that some berries experience predominant degradation (blackcurrant, rowanberry), whereas others benefit from enhanced extractability of phenolics (aronia, lingonberry, cranberry).

### Berries Skins Valorization Potential

Table 2 below summarizes the extent of phenolic retention in the solid fraction, identifies the major biochemical transformations occurring during fermentation, and evaluates the resulting valorization potential. This approach highlights species-specific patterns, showing whether primary fermentation acts predominantly as a degradative process or as a phenolic liberation step that enhances the economic and functional value of berry skins.

The species-dependent valorization profiles reveal that the economic and functional potential of fermented berry skins is shaped not only by the absolute retention of phenolics but also by the biochemical architecture of each berry’s phenolic matrix and its responsiveness to yeast-driven transformations. The fact that berries were dominated by highly polymerized or conjugated phenolics, such as cranberry and Aronia, demonstrates the greatest valorization potential because fermentation breaks down these structurally complex molecules into more extractable and bioaccessible forms. This shift toward low-molecular-weight acids and aglycones fundamentally increases the technological value of the skins, making them more suitable for applications where solubility, antioxidant efficacy, and processability are essential. In contrast, berries whose phenolic profiles rely heavily on labile compounds, such as flavan-3-ols and hydroxycinnamic acids in blackcurrant and rowanberry, experience extensive degradation during fermentation, indicating that their phenolic architecture is less resilient to oxidative and enzymatic stress. These losses suggest that their by-products may require modified fermentation regimes or protective strategies to maintain value. Lingonberry, positioned between these two extremes, illustrates how the balance between phenolic release and degradation can produce a mixed valorization pattern, where the breakdown of dominant catechins is countered by the emergence of simpler acids with higher stability and potential bioactivity. These findings collectively show that valorization is not determined solely by phenolic quantity but by the direction of molecular transformation during fermentation, which dictates whether the resulting phenolic profile becomes more functional, more extractable, and ultimately more suitable for incorporation into high-value food, cosmetic, or nutraceutical applications.

The phenolic profiles determined in berry skin pomaces revealed the presence of compound classes with well-established biological relevance. Hydroxycinnamic acids represented a substantial fraction of the quantified phenolics across species and fermentation states, reflecting their structural abundance in berry skins and their relative stability during processing. Flavan-3-ols, particularly catechin, dominated the profiles of non-fermented lingonberry and black currant skins, while fermentation resulted in a pronounced redistribution of this fraction. Flavonols and their glycosides, including quercetin- and kaempferol-based compounds, were consistently detected in Aronia, cranberry, and rowanberry skins, with fermentation promoting either enrichment or depletion depending on the molecular form. The appearance or increase in simple phenolic acids such as gallic, vanillic, and salicylic acids in fermented samples indicates hydrolysis or transformation of more complex phenolic precursors. These results demonstrate that berry skin pomaces retain a chemically diverse pool of phenolic compounds after juice processing and fermentation, with species-specific compositional shifts that may influence their functional properties and suitability for further valorization.

## 4. Discussion

Results from this study are broadly consistent with published data on berry phenolics and fermentation effects, and the absolute values obtained in this study fall within the ranges reported for similar matrices [46,47,48]. Recent publications confirm similar ranges for blackcurrant by-products. Herzyk et al. [49] reported total phenolic content in blackcurrant pomace extracts between 24 and 35 mg GAE/g DW, depending on pre-drying conditions and supercritical CO_2_ extraction efficiency. Untea et al. [50] obtained 12.4 mg GAE/g DW in fresh blackcurrant fruits and 21.8 mg GAE/g DW in pomace prior to digestion, both within the magnitude observed in our material. Likewise, Laczkó-Zöld et al. [51] found extractable polyphenols in blackcurrant residues ranging from 18.6 to 42.1 mg GAE/g DW, with antioxidant activity strongly associated with their high anthocyanin content. Blejan et al. [52] reported comparable levels in wild blackcurrant skins (20.3 mg GAE/g DW), confirming that skin-rich fractions comprise a major pool of berry phenolics. These data collectively demonstrate that the phenolic concentrations measured in our blackcurrant skins fall squarely within the published variability for similar berry processing by-products.

Fermentation affected these phenolic pools in a species-dependent manner that reflects both the dominant phenolic classes and their structural organization. In blackcurrant skins, total phenolics decreased by about two-thirds (−66%), driven mainly by strong reductions in sinapic and p-coumaric acids and catechin. This level of loss is comparable to or greater than that reported for other fermented berry matrices. For example, Markkinen et al. [53] reported 9–14% decreases in flavonols and 20–24% decreases in hydroxycinnamates during lactic fermentation of chokeberry juice, with anthocyanins remaining largely stable over the relatively short fermentation time. In our work, the much larger decline in blackcurrant skins suggests that prolonged primary alcoholic fermentation of solid fractions favours both oxidative and enzymatic degradation of cinnamic acids and flavan-3-ols and their diffusion into the liquid phase.

Lingonberry and rowanberry behaved similarly to blackcurrant, though with different dominant compounds. In lingonberry, catechin and p-coumaric acid were initially the major phenolics (≈5.1 and 4.4 mg/g DW, respectively), but both dropped sharply after fermentation (to 1.31 and 1.68 mg/g). This pattern indicates extensive loss of the principal hydroxycinnamates and flavan-3-ols, even though some hydroxybenzoic acids (e.g., salicylic, benzoic) and sinapic acid increased. Rowanberry skins also showed substantial decreases in key components such as caffeic acid, salicylic acid and quercetin-3-O-glucoside, with only a slight increase in sinapic acid. These findings support the view that fermentations in anthocyanin- and hydroxycinnamate-rich matrices tend to be degradation-dominated, in agreement with studies on cranberry and bilberry wines that reported final total phenolics below 500 mg/L, markedly lower than in the raw fruits [54,55,56].

Cranberry showed the most extensive liberation of low-molecular-weight phenolic acids among the studied species. In our samples, gallic acid was essentially absent in non-fermented skins but reached 1.96 mg/g DW after fermentation, becoming one of the dominant compounds, while quercetin glycosides and catechin declined. These observations are consistent with the solid-state fermentation study by Vattem and Shetty [57], who reported that fermentation of cranberry pomace strongly increased total extractable phenolics and DPPH activity and led to ellagic acid production, which was not detectable initially. They attributed such changes to fungal β-glucosidase activity and hydrolysis of ellagitannins and other conjugates. Our findings support a similar mechanism in cranberry skins: microbial enzymes likely convert high-molecular-weight tannins and ellagitannin derivatives into smaller phenolic acids such as gallic acid. Analogous partial preservation or enhancement of selected phenolics during fermentation has been observed in other berry residues. Meini et al. [58] showed that fermentation with *Aspergillus niger* promotes enzymatic transformation of phenolic compounds, including the release of simple phenolic acids such as gallic acid, highlighting that fermentation can simultaneously degrade complex phenolics while generating new extractable forms.

The behaviour of lingonberry and Aronia in our study also echoes observations from fermented composite products. Wang et al. [59] demonstrated that lactic fermentation of black chokeberry juice in a yogurt matrix yielded 3–4-fold higher total phenolic content (54 mg GAE/g DW) than control yogurt (~16 mg/g), suggesting that the combination of microbial enzymes and protein interactions enhances polyphenol extractability. In our system, although no dairy matrix was used, Aronia skins still showed increased flavonols, and cranberry skins gained new phenolic acids, indicating that alcoholic fermentation alone can be sufficient to mobilize bound phenolics. At the same time, not all systems respond similarly to microbial treatments. Markkinen et al. [53] reported that *Lactobacillus* strains could not effectively ferment pure lingonberry juice without enzyme pre-treatment, presumably because of lingonberry’s high benzoic acid content and associated antimicrobial activity. In our mixed fermentation system, lingonberry phenolics did change, but the overall trend was a decline in the major catechin and p-coumaric acid pool, with partial compensation through increases in simple phenolic acids. This suggests that for lingonberry, fermentation conditions and microbial strain selection are particularly critical if the goal is to enhance rather than diminish phenolic availability.

From a valorization perspective, these findings highlight the dual role of fermentation. For some berry by-products, primary fermentation is predominantly degenerative with respect to phenolic retention in the solid fraction, and strategies to mitigate oxidative and enzymatic losses (e.g., shorter fermentation, reduced oxygen exposure, targeted starter cultures) may be needed if skins are to be exploited as phenolic-rich ingredients. For other berries, notably Aronia and cranberry, fermentation can act as a processing step that enhances phenolic extractability and generates additional phenolic acids with potentially higher bioaccessibility. Our cross-species comparison under standardized conditions therefore provides a mechanistic and quantitative basis for designing species-appropriate fermentation and extraction strategies to maximize the phenolic value of berry skins in food, cosmetic, and nutraceutical applications.

## 5. Conclusions

Primary alcoholic fermentation influenced the phenolic composition of berry skins in a markedly species-dependent manner. In blackcurrant, lingonberry and rowanberry, fermentation resulted in substantial losses of total phenolics due to degradation and diffusion of dominant flavan-3-ols and hydroxycinnamates. Cranberry and chokeberry skins exhibited net increases in phenolic content, driven by enzymatic release of bound phenolics and formation of simple phenolic acids such as gallic acid. These results demonstrate that the initial phenolic architecture, free versus conjugated and polymeric forms, determines whether fermentation predominantly diminishes or enhances phenolic retention in the solid fraction.

From a valorization perspective, fermented skins of Aronia and cranberry retain high phenolic potential and therefore represent promising raw materials for the development of antioxidant ingredients in food or nutraceutical applications. In contrast, maximizing the functional value of blackcurrant, lingonberry and rowanberry skins may require modifications to processing conditions to minimize phenolic degradation, such as reduced oxygen exposure, shorter fermentation, or targeted enzyme or microbial treatments.

Despite providing a systematic comparison of phenolic transformations in berry skin fractions following primary fermentation, this study has several limitations. Only a single fermentation approach and microbial consortium were applied, and the observed changes therefore reflect species-specific responses under these defined conditions rather than the full range of possible fermentation outcomes. Future research should evaluate the effects of different microorganisms, including selected yeast and lactic acid bacteria strains, as well as controlled fermentation parameters, on phenolic stability, biotransformation pathways, and bioactivity. Such studies would enable optimization of fermentation strategies tailored to specific berry species and targeted functional properties.

This comparative evaluation provides new evidence that fermentation can serve either as a degradative step or as a phenolic liberation strategy depending on berry species. Selecting fermentation parameters according to the phenolic characteristics of the raw material will be essential for sustainable and efficient valorization of berry by-products generated in wine production.

## Figures and Tables

**Table 1:** Phenolic compound profile (µg/g) in five non-fermented and fermented berry species.

Compound	Black Currant (NF)	Black Currant (F)	Aronia (NF)	Aronia (F)	Lingonberries (NF)	Lingonberries (F)	Rowanberries (NF)	Rowanberries (F)	Cranberries (NF)	Cranberries (F)
Gallic acid	0.00 ± 0.00 c	0.00 ± 0.00 c	0.00 ± 0.00 c	0.00 ± 0.00 c	0.00 ± 0.00 c	0.00 ± 0.00 c	0.01 ± 0.00 b	0.01 ± 0.00 b	0.00 ± 0.00 c	1.96 ± 0.01 a
Epigallocatechin	0.00 ± 0.00 c	0.00 ± 0.00 c	0.00 ± 0.00 c	0.00 ± 0.00 c	0.00 ± 0.00 c	0.00 ± 0.00 c	0.06 ± 0.00 a	0.00 ± 0.00 c	0.00 ± 0.00 c	0.04 ± 0.00 b
Catechin	0.35 ± 0.03 d	0.11 ± 0.01 f	0.21 ± 0.00 e	0.00 ± 0.00 g	5.13 ± 0.03 a	1.31 ± 0.02 b	0.00 ± 0.00 g	0.00 ± 0.00 g	0.50 ± 0.02 c	0.00 ± 0.00 g
p-Hydroxybenzoic acid	0.00 ± 0.00 g	0.03 ± 0.00 c	0.03 ± 0.00 d	0.05 ± 0.00 a	0.00 ± 0.00 g	0.00 ± 0.00 g	0.03 ± 0.00 e	0.05 ± 0.00 b	0.00 ± 0.00 g	0.02 ± 0.00 f
Chlorogenic acid	0.15 ± 0.01 d	0.19 ± 0.00 c	0.40 ± 0.00 b	0.08 ± 0.00 f	0.51 ± 0.01 a	0.14 ± 0.00 d	0.00 ± 0.00 g	0.00 ± 0.00 g	0.10 ± 0.00 e	0.00 ± 0.00 g
Caffeic acid	0.09 ± 0.00 d	0.09 ± 0.00 d	0.19 ± 0.00 c	0.43 ± 0.00 b	0.10 ± 0.00 d	0.06 ± 0.00 e	0.50 ± 0.00 a	0.20 ± 0.01 c	0.00 ± 0.00 f	0.00 ± 0.00 f
p-Coumaric acid	0.99 ± 0.01 c	0.00 ± 0.00 e	0.00 ± 0.00 e	0.59 ± 0.00 d	4.35 ± 0.79 a	1.68 ± 0.40 b	0.00 ± 0.00 e	0.00 ± 0.00 e	0.00 ± 0.00 e	0.00 ± 0.00 e
Benzoic acid	0.51 ± 0.00 b	0.23 ± 0.01 d	0.02 ± 0.00 g	0.15 ± 0.01 e	0.09 ± 0.00 f	0.53 ± 0.00 a	0.00 ± 0.00 h	0.00 ± 0.00 h	0.30 ± 0.01 c	0.00 ± 0.00 h
Ferulic acid	0.00 ± 0.00 a	0.00 ± 0.00 a	0.00 ± 0.00 a	0.00 ± 0.00 a	0.00 ± 0.00 a	0.00 ± 0.00 a	0.00 ± 0.00 a	0.00 ± 0.00 a	0.00 ± 0.00 a	0.00 ± 0.00 a
Quercetin-3-O-rutinoside	0.00 ± 0.00 e	0.00 ± 0.00 e	0.00 ± 0.00 e	0.00 ± 0.00 e	0.00 ± 0.00 e	0.00 ± 0.00 e	0.04 ± 0.00 c	0.02 ± 0.00 d	0.40 ± 0.02 a	0.29 ± 0.00 b
Naringin	0.00 ± 0.00 a	0.00 ± 0.00 a	0.00 ± 0.00 a	0.00 ± 0.00 a	0.00 ± 0.00 a	0.00 ± 0.00 a	0.00 ± 0.00 a	0.00 ± 0.00 a	0.00 ± 0.00 a	0.00 ± 0.00 a
Vanillic acid	0.00 ± 0.00 c	0.00 ± 0.00 c	0.00 ± 0.00 c	0.00 ± 0.00 c	0.00 ± 0.00 c	0.00 ± 0.00 c	0.06 ± 0.00 a	0.03 ± 0.00 b	0.00 ± 0.00 c	0.03 ± 0.00 b
Salicylic acid	0.00 ± 0.00 h	0.11 ± 0.00 f	1.81 ± 0.00 a	1.06 ± 0.01 b	0.16 ± 0.00 f	0.84 ± 0.01 c	0.65 ± 0.00 d	0.30 ± 0.01 e	0.00 ± 0.00 h	0.08 ± 0.00 g
Sinapic acid	1.32 ± 0.01 a	0.28 ± 0.01 c	0.11 ± 0.00 e	0.25 ± 0.00 d	0.08 ± 0.00 f	0.43 ± 0.01 b	0.03 ± 0.00 g	0.09 ± 0.00 e	0.00 ± 0.00 h	0.09 ± 0.00 c
Ellagic acid	0.51 ± 0.00 a	0.00 ± 0.00 e	0.00 ± 0.00 e	0.00 ± 0.00 e	0.00 ± 0.00 e	0.00 ± 0.00 e	0.13 ± 0.00 d	0.00 ± 0.00 e	0.30 ± 0.01 b	0.19 ± 0.00 c
Kaempferol-3-O-glucoside	0.24 ± 0.00 c	0.08 ± 0.00 d	0.26 ± 0.00 b	0.95 ± 0.01 a	0.76 ± 0.02 b	0.00 ± 0.00 e	0.00 ± 0.00 e	0.00 ± 0.00 e	0.00 ± 0.00 e	0.00 ± 0.00 e
Myricetin	0.06 ± 0.00 d	0.02 ± 0.00 g	0.04 ± 0.00 f	0.02 ± 0.00 g	0.16 ± 0.00 a	0.13 ± 0.00 b	0.04 ± 0.00 e	0.02 ± 0.00 g	0.10 ± 0.00 c	0.04 ± 0.00 e
Valeric acid	0.13 ± 0.00 e	0.09 ± 0.00 e	0.44 ± 0.00 c	0.27 ± 0.01 d	3.85 ± 0.03 a	2.88 ± 0.03 b	0.11 ± 0.00 e	0.05 ± 0.00 f	0.00 ± 0.00 g	0.16 ± 0.00 e
trans-Cinnamic acid	0.16 ± 0.00 c	0.03 ± 0.00 f	0.24 ± 0.00 b	0.07 ± 0.00 e	0.00 ± 0.00 g	1.01 ± 0.01 a	0.08 ± 0.00 e	0.00 ± 0.00 g	0.00 ± 0.00 g	0.10 ± 0.00 d
Apigenin	0.00 ± 0.00 c	0.00 ± 0.00 c	0.00 ± 0.00 c	0.00 ± 0.00 c	0.00 ± 0.00 c	0.00 ± 0.00 c	0.07 ± 0.00 a	0.03 ± 0.00 b	0.00 ± 0.00 c	0.03 ± 0.00 b
Quercetin	0.07 ± 0.00 c	0.03 ± 0.00 e	0.21 ± 0.00 a	0.17 ± 0.00 b	0.00 ± 0.00 f	0.00 ± 0.00 f	0.04 ± 0.00 d	0.02 ± 0.00 e	0.00 ± 0.00 f	0.06 ± 0.00 d
Kaempferol	0.13 ± 0.00 d	0.31 ± 0.01 c	1.92 ± 0.01 b	2.98 ± 0.01 a	0.00 ± 0.00 e	0.00 ± 0.00 e	0.00 ± 0.00 e	0.00 ± 0.00 e	0.00 ± 0.00 e	0.00 ± 0.00 e
Quercetin-3-O-glucoside	0.09 ± 0.00 e	0.06 ± 0.00 e	0.36 ± 0.00 c	0.32 ± 0.00 c	0.00 ± 0.00 f	2.19 ± 0.01 a	0.54 ± 0.00 b	0.20 ± 0.01 d	0.50 ± 0.02 b	0.16 ± 0.00 d
Sum of all identified compounds	4.80	1.66	6.24	7.39	15.19	11.20	2.39	1.02	2.20	3.25

Note: Different lowercase letters within the same column indicate statistically significant differences between samples (*p* < 0.05).

**Table 2:** Valorization Potential of Berry Skin By-Products.

Berry Species	Key Phenolic Transformations	Resulting Valorization Potential	Valorization Rationale (Why?)
**Blackcurrant**	Loss of sinapic acid, p-coumaric acid, catechin; slight increase in chlorogenic acid	**Low**	Dominant phenolics are degraded or lost to the liquid phase, reducing extractable antioxidant capacity and limiting industrial value
**Chokeberry**	Increase in kaempferol, kaempferol-3-O-glucoside, caffeic and benzoic acids	**High**	Fermentation enhances the release of bound flavonols, improving suitability for functional ingredients and high-value antioxidant extracts
**Lingonberry**	Sharp decline in catechin and p-coumaric acid but increases in benzoic, salicylic and sinapic acids	**Moderate**	Despite losses in major flavan-3-ols, some liberated acids contribute to a moderate valorization potential, which could be improved by process optimization
**Rowanberry**	Reduction in caffeic acid, salicylic acid and quercetin-3-O-glucoside; slight increase in sinapic acid	**Low**	Fermentation leads mainly to degradation, leaving a limited phenolic pool for extraction-based valorization
**Cranberry**	Appearance of gallic acid (1.96 mg/g DW) and increases in simple phenolic acids	**Very High**	Fermentation liberates valuable low-molecular-weight phenolics, greatly increasing antioxidant potential and suitability for nutraceutical use

## Data Availability

The original contributions presented in this study are included in the article/Supplementary Materials. Further inquiries can be directed to the corresponding author.

## Supplementary Materials

The following supporting information can be downloaded at: https://www.mdpi.com/article/10.3390/plants15020296/s1, Figure S1: The chromatogram for non fermented black currant. Following numbers mean separated phenolic compounds: (1) catechin, (2) chlorogenic, (3) caffeic, (4) p-coumaric, (5) benzoic, (6) sinapic, (7) ellagic, (8) kaempferol-3-O-glucosiode, (9) myricetin, (10) t-cinaminic, (11) quercetin, (12) valeric, (13) kaempferol, (14) quercetin-3-O-glucoside; Figure S2: The chromatogram for fermented black currant. Following numbers mean separated phenolic compounds: (1) catechin, (2) chlorogenic, (3) caffeic, (4) p-coumaric, (5) benzoic, (6) sinapic, (7) kaempferol-3-O-glucosiode, (8) myricetin, (9) valeric, (10) t-cinaminic, (11) quercetin, (12) kaempferol, (13) quercetin-3-O-glucoside; Figure S3: The chromatogram for non fermented aronia. Following numbers mean separated phenolic compounds: (1) catechin, (2) p-hydrobenzoic, (3) chlorogenic, (4) caffeic, (5) benzoic, (6) salicylic, (7) sinapic, (8) kaempferol-3-O-glucosiode, (9) myricitin, (10) valeric, (11) t-cinaminic, (12) quercetin, (13) kaempferol, (14) quercetin-3-O-glucoside; Figure S4: The chromatogram for non-fermented aronia. Following numbers mean separated phenolic compounds: (1) catechin, (2) p-hydrobenzoic, (3) chlorogenic, (4) caffeic, (5) benzoic, (6) salicylic, (7) sinapic, (8) kaempferol-3-O-glucosiode, (9) myricitin, (10) valeric, (11) t-cinaminic, (12) quercetin, (13) kaempferol, (14) quercetin-3-O-glucoside; Figure S5: The chromatogram for fermented aronia. Following numbers mean separated phenolic compounds: (1) chlorogenic, (2) p-hydrobensoic, (3) caffeic, (4) p-coumaric, (5) benzoic, (6) salicylic, (7) sinapic, (8) kaempferol-3-O-glucosiode, (9) myricetin, (10) t-cinaminic, (11) quercetin, (12) valeric, (13) kaempferol, (14) quercetin-3-O-glucoside; Figure S6: The chromatogram for fermented lingoberries. Following numbers mean separated phenolic compounds: (1) catechin, (2) chlorogenic, (3) caffeic, (4) p-coumaric, (5) benzoic, (6) salicilic, (7) sinapic, (8) kaempferol-3-O-glucosiode, (9) myrycetin, (10) valeric; Figure S7: The chromatogram for fermented lingoberries. Following numbers mean separated phenolic compounds: (1) catechin, (2) chlorogenic, (3) caffeic, (4) p-coumaric, (5) benzoic, (6) salicylic, (7) sinapic, (8) myrycetin, (9) t-cinaminic, (10) valeric, (11) quercetin-3-O-glucoside; Figure S8: The chromatogram for cranberries. Following numbers mean separated phenolic compounds: (1) catechin, (2) chlorogenic, (3) p-coumaric, (4) benzoic, (5) salicylic, (6) sinapic, (7) kaempferol-3-O-glucosiode, (8) myrycetin, (9) t-cinaminic, (10) quercetin, (11) valeric, (12) quercetin-3-O-glucoside; Figure S9: The chromatogram for non fermented rowanberries. Following numbers mean separated phenolic compounds: (1) gallic, (2) p-hydrobensoic, (3) p-coumaric, (4) ferulic, (5) salicylic, (6) quercetine-3-O-rutinoside, (7) sinapic, (8) kaempferol-3-O-glucosiode, (9) myrycetin, (10) t-cinaminic, (11) valeric, (12) apigenin, (13) quercetin-3-O-glucoside; Figure S10: The chromatogram for fermented rowanberries. Following numbers mean separated phenolic compounds: (1) gallic, (2) epigallocatechin, (3) p-hydrobensoic, (4) caffeic, (5) vanilic, (6) salicilic, (7) quercetin-3-O-rutinoside (8) sinapic, (9) ellagic, (10) myrycetin, (11) t-cinaminic, (12) valeric, (13) apigenin, (14) quercetin, (15) quercetin-3-O-glucoside.
