# Supplementary material for: Primary Fermentation in Wine Production Influence on Phenolic Retention and Valorization Potential of Berry Skin By-Products
Source: Plants (Basel). 2026 Jan 19;15(2):296. doi: 10.3390/plants15020296 (PMC12845066; doi:10.3390/plants15020296)
Supplement: Supplementary file 1 [file plants-15-00296-s001.zip › plants-4051999-supplementary.pdf]

Chromatograms of analyzed berries

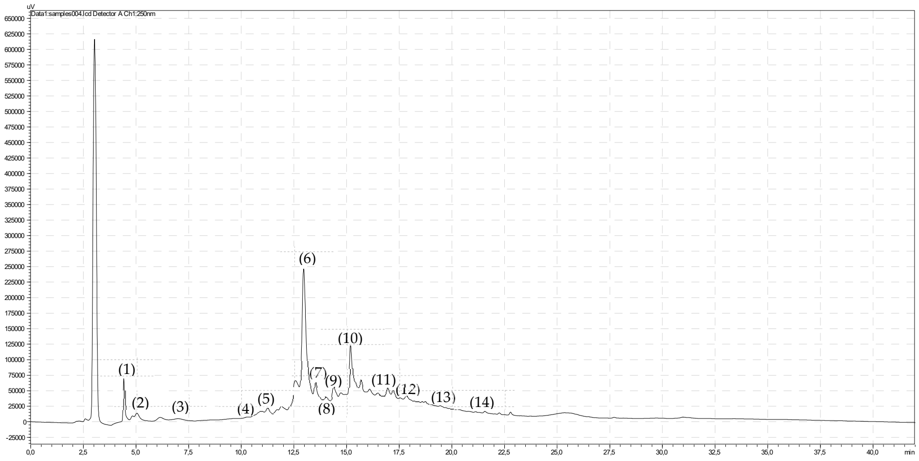

Figure S1. The chromatogram for non fermented black currant. Following numbers mean separated phenolic compounds: (1) catechin, (2) chlorogenic, (3) caffeic, (4) p-coumaric, (5) benzoic, (6) sinapic, (7) ellagic, (8) kaempferol-3-O-glucosiode, (9) myricetin, (10) t-cinaminic, (11) quercetin, (12) valeric, (13) kaempferol, (14) quercetin-3-O-glucoside

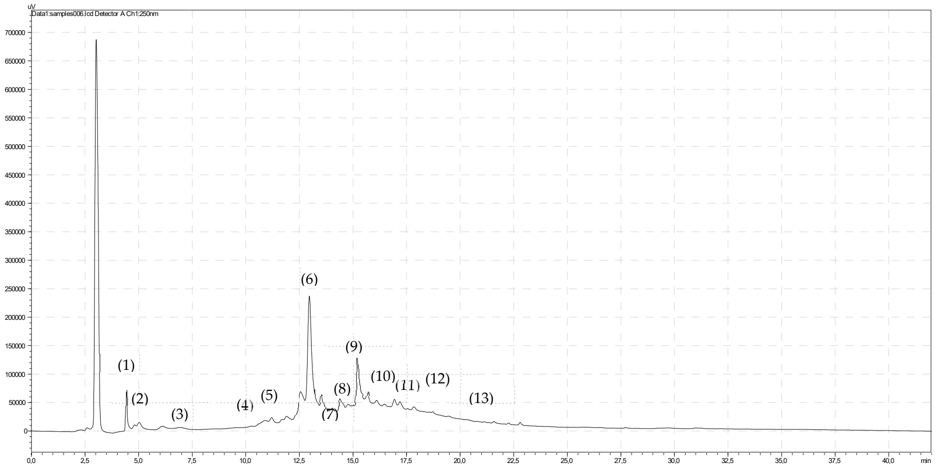

Figure S2. The chromatogram for fermented black currant. Following numbers mean separated phenolic compounds: (1) catechin, (2) chlorogenic, (3) caffeic, (4) p-coumaric, (5) benzoic, (6) sinapic, (7) kaempferol-3-O-glucosiode, (8) myricetin, (9) valeric, (10) t-cinaminic, (11) quercetin, (12) kaempferol, (13) quercetin-3-O-glucoside

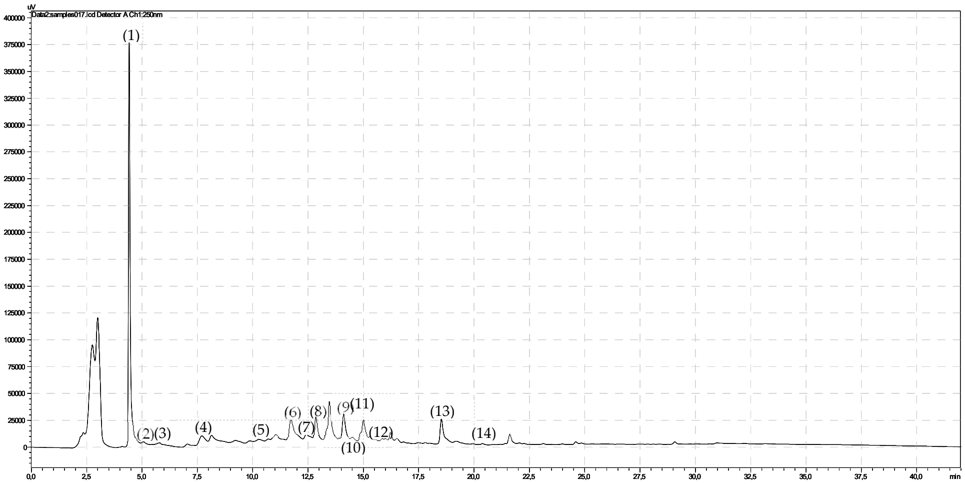

Figure S3. The chromatogram for non fermented aronia. Following numbers mean separated phenolic compounds: (1) catechin, (2) p-hydrobenzoic, (3) chlorogenic, (4) caffeic, (5) benzoic, (6) salicylic, (7) sinapic, (8) kaempferol-3-O-glucosiode, (9) myricitin, (10) valeric, (11) t-cinaminic, (12) quercetin, (13) kaempferol, (14) quercetin-3-O-glucoside.

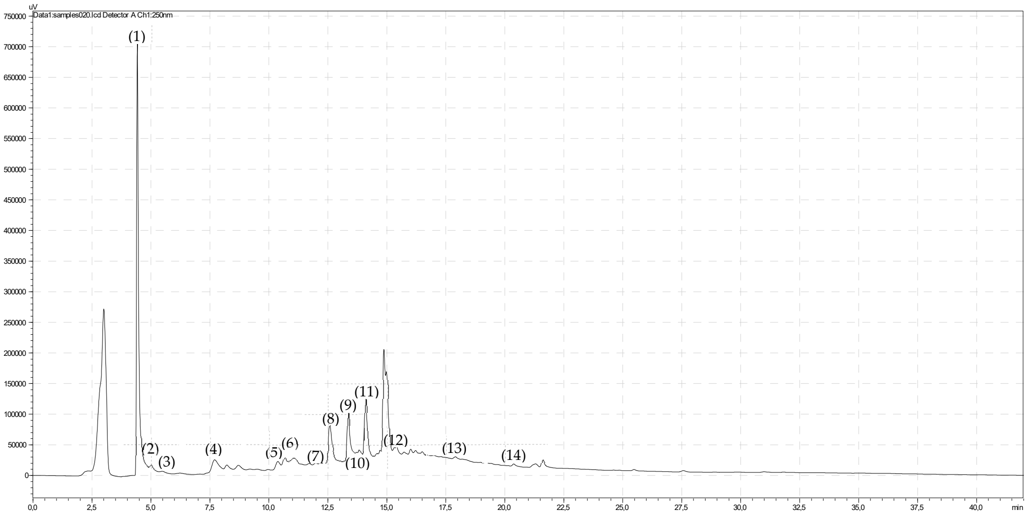

Figure S4. The chromatogram for non-fermented aronia. Following numbers mean separated phenolic compounds: (1) catechin, (2) p-hydrobenzoic, (3) chlorogenic, (4) caffeic, (5) benzoic, (6) salicylic, (7) sinapic, (8) kaempferol-3-O-glucosiode, (9) myricitin, (10) valeric, (11) t-cinaminic, (12) quercetin, (13) kaempferol, (14) quercetin-3-O-glucoside.

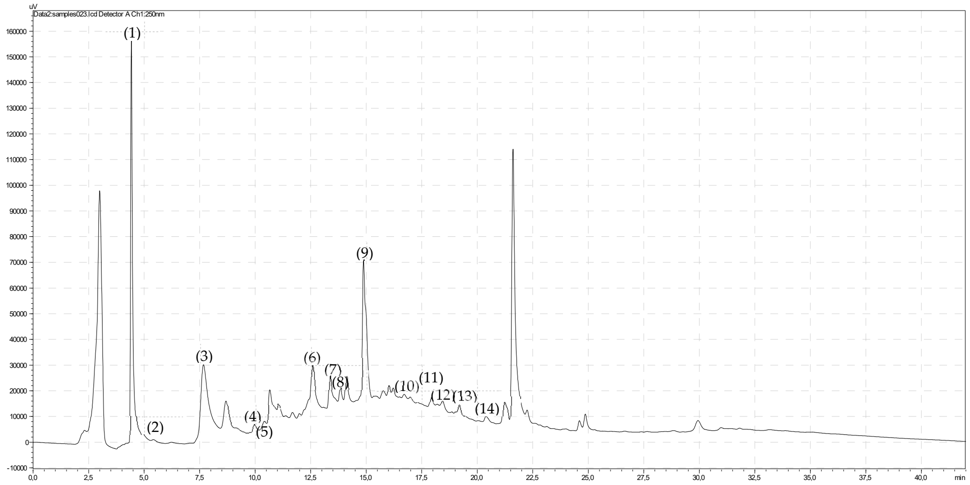

Figure S5. The chromatogram for fermented aronia. Following numbers mean separated phenolic compounds: (1) chlorogenic, (2) p-hydrobensoic, (3) caffeic, (4) p-coumaric, (5) benzoic, (6) salicylic, (7) sinapic, (8) kaempferol-3-O-glucosiode, (9) myricetin, (10) t-cinaminic, (11) quercetin, (12) valeric, (13) kaempferol, (14) quercetin-3-O-glucoside.

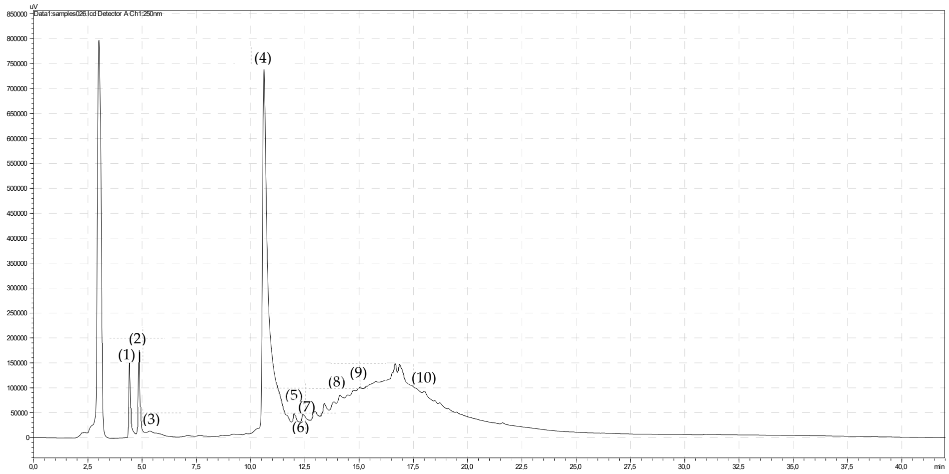

Figure S6. The chromatogram for fermented lingoberries. Following numbers mean separated phenolic compounds: (1) catechin, (2) chlorogenic, (3) caffeic, (4) p-coumaric, (5) benzoic, (6) salicilic, (7) sinapic, (8) kaempferol-3-O-glucosiode, (9) myrycetin, (10) valeric.

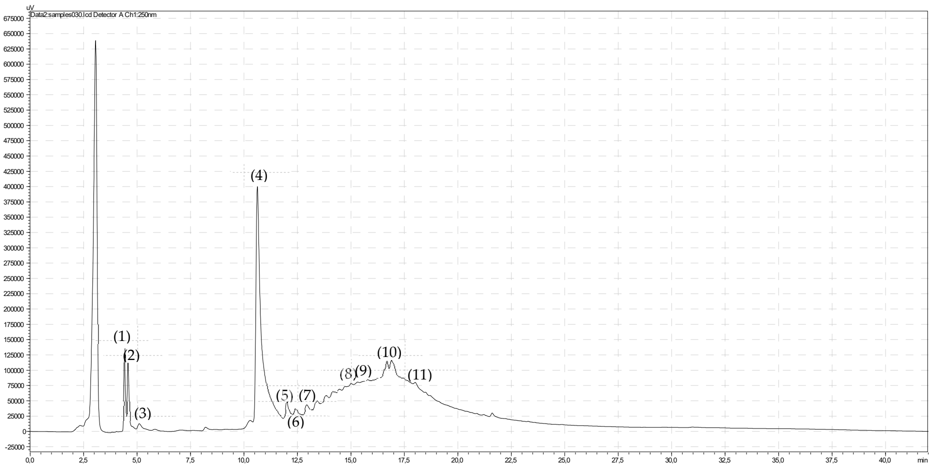

Figure S7. The chromatogram for fermented lingoberries. Following numbers mean separated phenolic compounds: (1) catechin, (2) chlorogenic, (3) caffeic, (4) p-coumaric, (5) benzoic, (6) salicylic, (7) sinapic, (8) myrycetin, (9) t-cinaminic, (10) valeric, (11) quercetin-3-O-glucoside

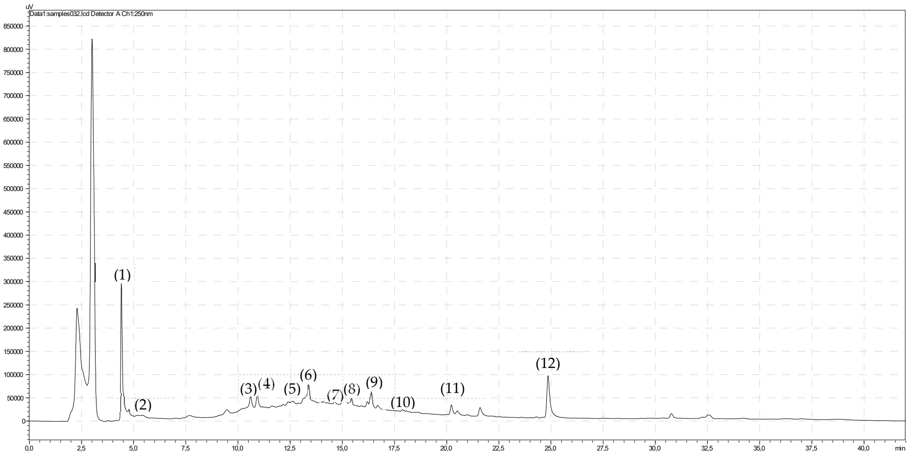

Figure S8. The chromatogram for cranberries. Following numbers mean separated phenolic compounds: (1) catechin, (2) chlorogenic, (3) p-coumaric, (4) benzoic, (5) salicylic, (6) sinapic, (7) kaempferol-3-O-glucosiode, (8) myrycetin, (9) t-cinaminic, (10) quercetin, (11) valeric, (12) quercetin-3-O-glucoside

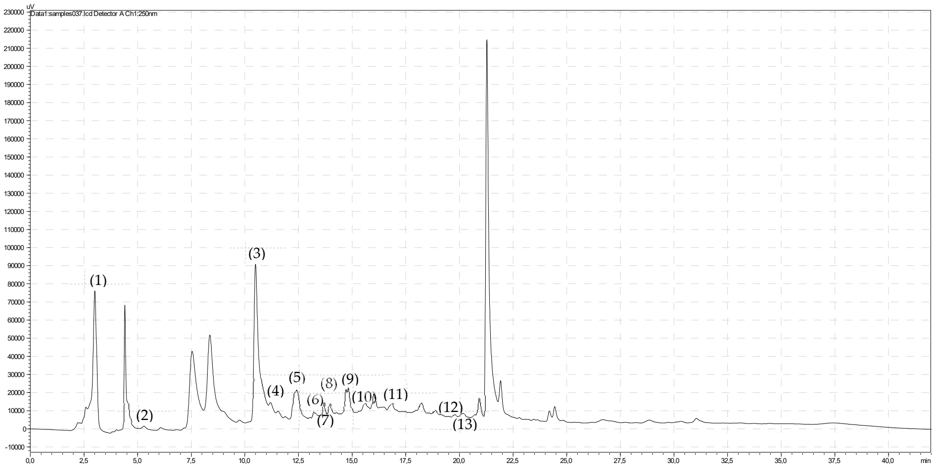

Figure S9. The chromatogram for non fermented rowanberries. Following numbers mean separated phenolic compounds: (1) gallic, (2) p-hydrobensoic, (3) p-coumaric, (4) ferulic, (5) salicylic, (6) quercetine-3-O-rutinoside, (7) sinapic, (8) kaempferol-3-O-glucosiode, (9) myrycetin, (10) t-cinaminic, (11) valeric, (12) apigenin, (13) quercetin-3-O-glucoside

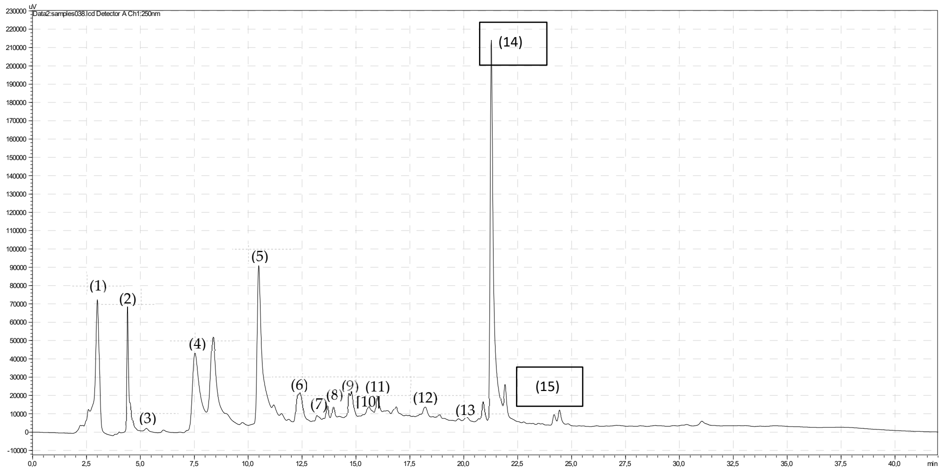

Figure S10. The chromatogram for fermented rowanberries. Following numbers mean separated phenolic compounds: (1) gallic, (2) epigallocatechin, (3) p-hydrobensoic, (4) caffeic, (5) vanilic, (6) salicilic, (7) quercetin-3-O-rutinoside (8) sinapic, (9) ellagic, (10) myrycetin, (11) t-cinaminic, (12) valeric, (13) apigenin, (14) quercetin, (15) quercetin-3-O-glucoside
